# What Clinical Trials Are Needed for Treatment of Leiomyosarcoma?

**DOI:** 10.1007/s11864-021-00928-y

**Published:** 2022-03-11

**Authors:** Bernd Kasper, Lorenzo D’Ambrosio, Elizabeth J. Davis, Matthew Ingham, Javier Martin Broto, Jonathan C. Trent, Winan J. van Houdt, Brian A. Van Tine

**Affiliations:** 1 Mannheim University Medical Center, University of Heidelberg, Theodor-Kutzer-Ufer 1-3, D-68167 Mannheim, Germany; 2 Department of Oncology, University of Turin, Turin, Italy; 3 Department of Internal Medicine, Division of Hematology/Oncology, Vanderbilt University Medical Center, Nashville, USA; 4 Columbia University School of Medicine, New York, USA; 5 Medical Oncology Department, University Hospital Fundacion Jimenez Diaz, Madrid, Spain; 6 Sylvester Comprehensive Cancer Center, University of Miami, Miami, USA; 7 Department of Surgical Oncology, The Netherlands Cancer Institute, Amsterdam, The Netherlands; 8 Siteman Cancer Center, Washington University in St. Louis, St. Louis, USA

**Keywords:** Leiomyosarcoma, Clinical trials, NLMSF, Treatment, Research

## Abstract

Leiomyosarcoma is one of the most common subtypes of soft tissue sarcomas accounting for approximately 20% of sarcomas. As leiomyosarcoma patients frequently develop metastatic disease, effective systemic therapies are needed to improve clinical outcomes. The overall activity of the currently available conventional systemic therapies and the prognosis of patients with advanced and/or metastatic disease are poor. As such, the treatment of this patient population remains challenging. As a result, there is a clear unmet medical need, and designing and performing meaningful clinical studies are of utmost importance to improve the prognosis of this patient group. Therefore, the aim of this review is to briefly summarize state-of-the-art treatments for leiomyosarcoma patients and to describe trial characteristics needed for informative clinical studies.

## Introduction

Soft tissue sarcomas (STS) represent a highly heterogeneous group of mesenchymal malignancies comprising more than 150 histological subtypes. Leiomyosarcoma (LMS) is one of the most frequent subtypes accounting for approximately 20% of patients. LMS occurs in middle-aged or older adults with a female predominance. LMS originates from the smooth muscle or their precursor cells, and thus can arise anywhere in the body with a predilection for the retroperitoneum, the extremities, and the uterus [1]. LMS can be divided into “extra-uterine” (retroperitoneal, gastrointestinal, extremity, or subcutaneous) and “uterine” LMS, each with distinct clinicopathological characteristics [2, 3]. Diagnosis and staging of patients with LMS are in line with the general recommendations for STS and visceral sarcomas [4] and overall management of LMS patients should be part of a multidisciplinary team in a high-volume sarcoma reference center. Despite complete resection of the primary tumor, LMS patients frequently develop metastatic disease; therefore, effective systemic therapies are needed. However, the overall activity of the currently available conventional systemic therapies and the prognosis of patients with advanced or metastatic disease are still poor, making the treatment of LMS patients challenging. Having clearly identified an unmet medical need, designing and performing meaningful clinical studies are of utmost importance to improve the prognosis of this patient population. Therefore, the aim of this review is to briefly summarize state-of-the-art treatments for LMS patients and to describe trial characteristics for the optimal design of clinical studies in this patient group. This work is based on a recent joint white paper from the National LeioMyoSarcoma Foundation (NLMSF) in collaboration with Sarcoma Patients EuroNet (SPAEN) and has been supported by the NLMSF [5••].

## Treatment paradigms for leiomyosarcoma patients

Surgery remains the cornerstone in the management of patients with localized LMS and the standard surgical procedure is a wide excision with negative margins (R0) [4]. In the case of R1 or R2 resections, re-operation in experienced centers is considered following possible preoperative treatments. Significant independent predictors for local recurrence are size and margin, whereas predictors for distant recurrence are size and grade [6]. In patients with extremity high-risk LMS (G2-3, deep ≥ 5 cm lesions), adjuvant or neoadjuvant radiation therapy is administered in addition to surgery. In patients with retroperitoneal and pelvic LMS, especially if low grade and borderline resectable, consideration should be given to neoadjuvant radiation based upon the results of the European Organisation for Research and Treatment of Cancer (EORTC) STRASS trial [7•]. Adjuvant chemotherapy is not globally accepted as the standard treatment strategy for the postoperative therapy of adult patients with LMS but can be considered in high-risk patients to reduce the risk of local recurrence and increase survival rates [4]. Neoadjuvant chemotherapy may have the same potential benefits as adjuvant chemotherapy, but similarly universal consensus does not exist. Neoadjuvant chemotherapy does have the advantage to allow for early response evaluation, to potentially prevent subsequent adjuvant chemotherapy, to treat micro-metastatic disease, and to downsize tumors allowing for less extensive surgical procedures. As of today, neoadjuvant chemotherapy as well as radiation therapy may be considered for patients with high-risk extremity/trunk LMS (lesion diameter ≥ 5 cm, tumor deep to fascia, adjacent to bone or neurovascular structures, invasion of skin, or based on prediction models such as Sarculator) [8]. The efficacy of neoadjuvant chemotherapy in retroperitoneal LMS (and liposarcomas) is currently being evaluated in the EORTC/Soft Tissue and Bone Sarcoma Group (STBSG) STRASS-2 trial in patients with resectable retroperitoneal sarcomas (NCT04031677), which hopefully may settle the long-lasting controversial debate about this topic. Unfortunately, there are no biomarkers available predicting responses to the different neoadjuvant chemotherapy regimens, preventing optimized patient selection for perioperative treatment strategies.

Standard first-line chemotherapy for STS consists of anthracycline-based regimens, and doxorubicin is the first-line chemotherapy of choice in patients with advanced LMS [4]. Doxorubicin plus ifosfamide demonstrated a significantly higher response rate and longer progression-free survival (PFS) compared to single-agent doxorubicin, but no significant difference in overall survival (OS) in a trial including all STS subtypes [9•]. Interestingly, the addition of ifosfamide was not found to be beneficial in the LMS subgroup in a post hoc analysis of this trial. In patients with LMS, the combination of doxorubicin plus dacarbazine is another option for multi-agent first-line chemotherapy [10]. Although ifosfamide might still retain some efficacy in women with uterine LMS, it appears to be less effective for patients with extra-uterine LMS [11, 12]. In a randomized phase 3 trial in first-line advanced STS, no significant difference in response rate, PFS, and OS was observed between single-agent doxorubicin and gemcitabine plus docetaxel, although doxorubicin was better tolerated with similar findings for the LMS cohort [13]. Promising data have been reported for the first-line combination of doxorubicin plus trabectedin in LMS [14]; however, final results from the randomized phase 3 trial comparing this combination versus doxorubicin alone are awaited (NCT02997358). In second line or later, trabectedin is a standard option for the treatment of advanced STS (including LMS) after failure of doxorubicin with or without ifosfamide, or for patients “unsuited” to receive these agents. Chemosensitivity to trabectedin has been noted in different STS subtypes, but best responses have been observed in LMS and liposarcomas [15, 16, 17]. Dacarbazine is a reasonable choice to consider in the refractory setting for LMS, and can be combined with gemcitabine. This combination is generally well tolerated and given on a convenient schedule [18]. Additionally, uterine LMS has an unusual sensitivity to dacarbazine. Two randomized studies comparing the efficacy of gemcitabine plus docetaxel versus gemcitabine alone reported divergent findings in patients with relapsed or metastatic LMS [19, 20]. In a subsequent pooled analysis, no significant improvement of response rate and PFS could be demonstrated by the addition of docetaxel for LMS [21]. Pazopanib is recommended for selected subtypes of advanced STS including LMS after prior chemotherapy for advanced and/or metastatic disease. The PALETTE trial included 165 patients with LMS. Pazopanib was shown to significantly prolong PFS; however, this did not translate into a statistically significant OS difference compared to placebo [22, 23]. It should be highlighted that the phase 3 eribulin trial included LMS and liposarcoma patients. Interestingly, higher response rates and rates of disease control were seen with dacarbazine for the LMS cohort in comparison to liposarcoma patients; this may have been the reason that eribulin was deemed ineffective for the LMS population [24]. Table 1 illustrates key studies on the current management of advanced/metastatic patients with STS/LMS.

**Table 1:** Key studies on current clinical management of advanced/metastatic STS/LMS

Agent(s)	Phase	*n*	Line	ORR		PFS (months)		OS (months)	
Doxorubicin vs doxorubicin + ifosfamide [9•]	III	455	1st	14%	26%	4.6	7.4	12.8	14.3
Doxorubicin vs gemcitabine + docetaxel [13]	III	257	1st	19%	20%	5.4	5.5	17.6	15.5
Gemcitabine vs gemcitabine + docetaxel [19]	II	122	1st–3rd	8%	16%	3.0	6.2	11.5	17.9
Dacarbazine vs gemcitabine + dacarbazine [18]	II	113	2nd+	25%^a^	49%^a^	2	4.2	8.2	16.8
Pazopanib vs placebo [22]	III	372	2nd+	6%	0%	4.6	1.6	12.5	10.7
Gemcitabine + docetaxel [25]	II	45	1st	25%		7.1		17.9	
Trabectedin vs dacarbazine [26]	III	403	3rd+	10%	7%	4.8	1.5	14.1	13.6
Temozolomide [27]	II	60	3rd+	9%	15%	2.3		13.8	

*ORR*, overall response rate; *PFS*, progression-free survival; *OS*, overall survival. ^a^Clinical benefit rate including stable diseases

## What clinical trials are needed for LMS?

The overall effectiveness of the currently available systemic treatment options for patients with LMS in the advanced and/or metastatic setting is limited; thus, patients’ overall prognosis remains poor. Therefore, designing and performing clinically meaningful and promising studies are of utmost importance to improve the prognosis of this patient population. The aim of this section is to describe trial characteristics for designing effective clinical studies in this distinct patient group.

1. *Studies should be LMS-specific*: Evidence-based data for LMS mainly comes from clinical trials open for the recruitment of a variety of heterogeneous STS subtypes; there are few prospective trials exclusively designed for the inclusion of LMS or even uterine LMS patients. Here are a few positive examples: (1) The North Eastern German Society of Gynaecological Oncology is currently evaluating the role of pazopanib versus pazopanib plus gemcitabine in the treatment of advanced or metastatic uterine LMS in an ongoing prospective randomized controlled phase 2 trial (PazoDoble; NCT02203760). (2) The French Sarcoma Group has conducted a randomized phase 3 study comparing the efficacy of doxorubicin plus trabectedin followed by trabectedin versus doxorubicin alone in LMS patients; final results are eagerly awaited (LMS-04; NCT02997358). (3) The EORTC/STBSG is currently developing an open label, randomized, phase 2 study on doxorubicin, doxorubicin plus dacarbazine, or gemcitabine plus dacarbazine for first-line treatment of advanced LMS patients (DODECANESO) based on a retrospective STBSG analysis [28]. Without doubt, international collaboration is essential to perform LMS-specific trials. The importance of including reference centers and reference networks for recruiting more patients into clinical trials is critical in this context.
2. *Studies should focus on certain clinical settings*: The majority of clinical studies are currently being conducted in the metastatic disease setting, mainly in later treatment lines (3rd/4th/5th line) potentially prolonging patients’ lives for only a few months. Other important scenarios where clinical trials are needed include the following: (1) Performing clinical studies in the neoadjuvant setting especially in high-risk localized LMS has the potential to actually cure patients, if the appropriate perioperative systemic regimen is administered. (2) When performing clinical studies in the (neo-)adjuvant setting, biomarkers are needed for response prediction as described below in more detail. Moreover, this is also the case for the metastatic disease setting. (3) The potential of performing “window-of-opportunity” studies should be emphasized to allow for fast response evaluation, to analyze biological processes, and to include more patients into clinical studies. Patients that are undergoing surgery, either in the primary, locally recurrent, or even metastatic setting, are excellent candidates to study new drugs or drug combinations with the opportunity to study both radiological and pathological mechanisms of response and resistance.
3. *Studies should explore new therapeutic avenues*: Besides the evaluation of the activity of conventional chemotherapeutic agents for LMS, new treatment avenues need to be explored. There are a number of ongoing trials exploring the possible value of immunotherapy in STS [29], including anti-PD1/PD-L1 monotherapy [30, 31], combined PD1/CTLA4 inhibition [32], or PD1 therapy combined with cyclophosphamide [33] or anti-VEGF tyrosine kinase inhibitor (TKI) axitinib [34], although the numbers of LMS patients included in these all-comer studies are small. Obviously, single-agent PD1 blockade does not seem to be the optimal LMS strategy, but hopefully combination therapies with other agents will be more promising. Multiple retrospective studies have suggested that STS/LMS do have underlying immunogenicity [35, 36, 37], but the exact therapeutic strategy to exploit this remains elusive. A large study of ~1000 LMS tissue samples suggests in a very small number of patients tumors harbor classic immunotherapy response markers. Additionally, this study found most tumor microenvironments had markers associated with low T cell but high for fibroblast abundance. This observation suggests clinical trials for LMS patients should include strategies to increase T cell abundance in the tumor microenvironment [38]. Ongoing clinical trials are combining cytotoxic chemotherapy, including doxorubicin, gemcitabine, and trabectedin, with checkpoint blockade, which may help to increase tumor immunogenicity of “cold” tumors: (1) A phase 2 study from the German Interdisciplinary Sarcoma Group (GISG) testing the combined treatment with nivolumab plus trabectedin in patients with metastatic or inoperable STS has a dedicated LMS cohort (GISG-15; NiTraSarc; NCT03590210). (2) Cabozantinib is being explored in a randomized study with or without dual PD1/CTLA4 checkpoint blockade, with a broader spectrum TKI potentially more impactful to the tumor microenvironment than narrow VEGF inhibitors (NCT04551430). (3) Anlotinib is being evaluated in a randomized phase 3 trial with a specific LMS cohort (APROMISS; NCT03016819).
4. Studies should follow a clear biological rationale: Based on recent research suggesting that LMS may harbor characteristic defects in the homologous recombination DNA repair pathway [39••, 40, 41, 42], a number of trials are currently evaluating PARP inhibitor–based approaches: (1) One trial is evaluating olaparib plus trabectedin versus doctor’s choice in various solid tumors harboring deficiency in DNA repair but is not sarcoma-specific (GISG-16; TopArt; NCT03127215). (2) A phase 1B trial of the combination of olaparib plus trabectedin in patients with previously treated advanced/metastatic STS has shown activity especially in LMS patients [43]. A phase 2 randomized study comparing standard trabectedin versus the combination of trabectedin plus olaparib is currently ongoing with a dedicated stratification for L-sarcomas (NCT03838744). A phase 2 single-arm trial of the same treatment combination in patients with advanced sarcomas has a LMS-specific cohort (NCT04076579). (3) Another phase 2 study is testing the combination of olaparib plus temozolomide specifically in patients with advanced metastatic or unresectable uterine LMS (NCT03880019) and could demonstrate promising results with an overall response rate of 27%, a median PFS of 6.9 months, and a median duration of response of 12 months [44], a perfect example for a successful bench-to-bedside approach. In this context, correlative studies are critical such as the example of three current GEIS (Spanish Sarcoma Research Group) studies in a selected group of STS histologies including LMS: In an upfront phase 2 trial, the compound LB100 will be explored in combination with doxorubicin versus doxorubicin alone in advanced L-sarcomas. In a second line trial, selinexor is combined with gemcitabine in a LMS-specific cohort (NCT04595994). Additionally, LMS patients will be enrolled in a new cohort of IMMUNOSARC-2 exploring immune mechanisms of tumor cell death for the combination of doxorubicin, dacarbazine, and nivolumab (NCT03277924). For all these trials, correlative studies with compulsory tumor blocks at baseline will be performed.
5. *Studies should evaluate the role of biomarkers*: Circulating tumor DNA (ctDNA) offers a rapid and noninvasive method of next-generation sequencing (NGS) that could be used for diagnosis, prognostic assessment, disease-response assessment to therapy, and detection of recurrence [45, 46, 47]. This strategy is worth exploring also in tumors not harboring a clear-cut gene driver like LMS. NGS of ctDNA allows identification of somatic and potentially germline genomic alterations in plasma from LMS patients [48, 49]; however, further validation and prospective evaluation are warranted to investigate the clinical utility of ctDNA especially for LMS patients: (1) A Sarcoma Alliance for Research Through Collaboration (SARC)–funded pilot study is evaluating ctDNA as a biomarker of relapse-free survival and response to therapy in patients with high-grade, high-risk, localized LMS. (2) A SARC-supported study of ctDNA as biomarker of sarcoma response to chemotherapy in patients with metastatic LMS is currently being planned. (3) Perhaps a molecular “signature” could serve as a better prognostic and predictive biomarker than the anatomic location. Data from several retrospective studies in LMS have shown that the Complexity INdex in SARComas (CINSARC) has utility in predicting risk of relapse [50, 51]. CINSARC is currently undergoing prospective evaluation in the perioperative setting (NCT03805022, NCT02789384, and NCT04307277).
6. *Studies should capture Patient-Reported Outcomes (PROs)*: There is growing recognition of the potential value offered by PROs fostered by patient involvement in clinical research. The work to develop a multidimensional sarcoma-specific scale is underway; however, there is some distance still to go to have a LMS-specific one. Validated composite tools to gather multidimensional data which enable a Health-Related Quality-of-Life (HRQoL) to be assessed are available with the weakness that they measure a “moment in time” rather than give a full picture of patient experience. It is now possible to construct questionnaires exploring detailed aspects of the patient experience opening to individual PROs. Item libraries are available such as the one of the EORTC Quality of Life Group containing over 900 PRO items, each of them in many languages and validated [52]. An important development has been the PRO Common Terminology Criteria for Adverse Events (CTCAE) from NCI [53]. The CTCAE has been a mainstay of cancer clinical trial practice and reporting for many years, but the grading relies on clinician observation of patients’ experience. The PRO version calls for patients to report their experience first-hand. Gathering these data using smartphones and internet reporting opens the way for a more sensitive and often more accurate reporting of adverse events in clinical studies.

## Conclusions

**Fig. 1:**
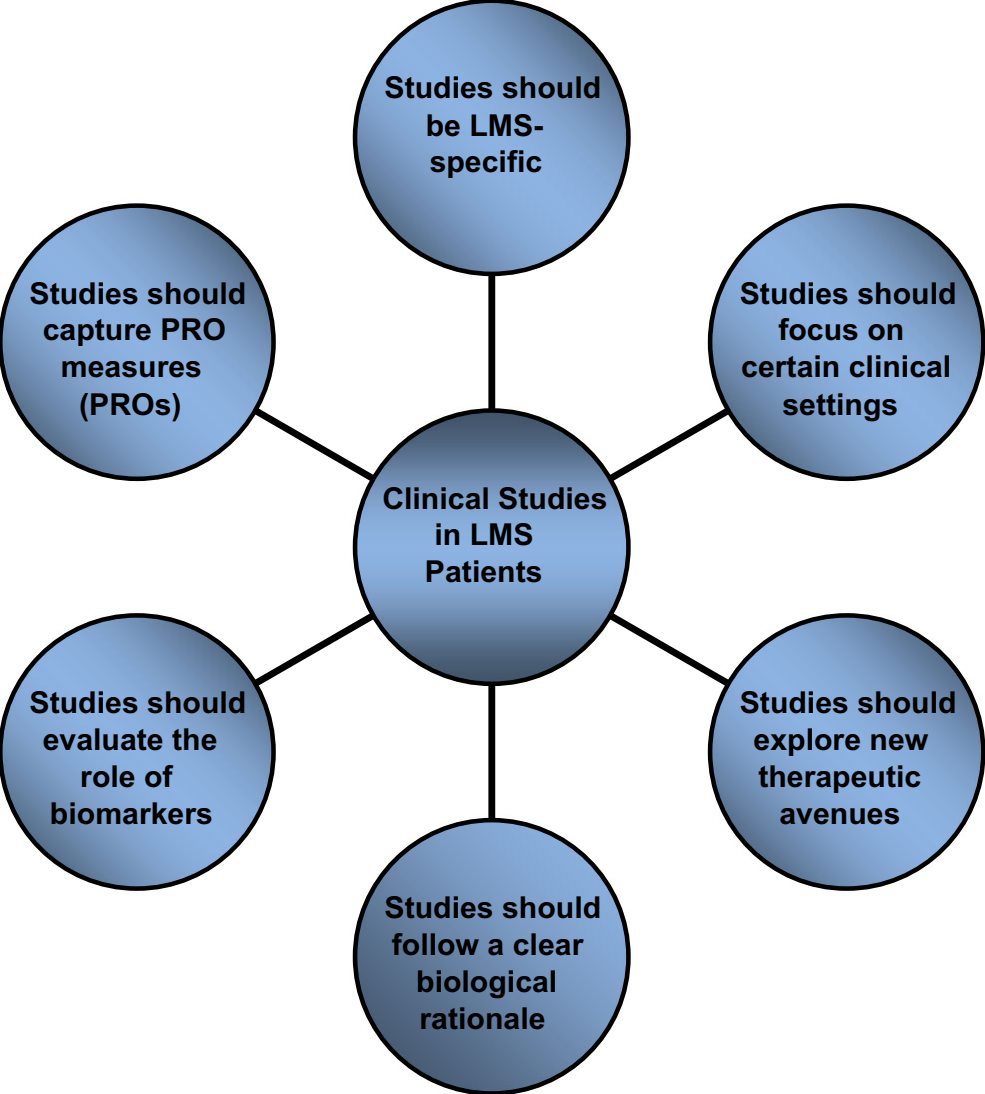
Trial characteristics for designing clinical studies in LMS.

In summary, there is a clear need for large international randomized or singlearm LMS-specific clinical trials, with an underlying biological rationale. It is strongly advisable to seek therapeutic advice of a high-volume reference center or to enroll patients in suitable subtype-specific clinical studies, factors clearly linked to a superior outcome for this patient group [54, 55]. Figure 1 summarizes trial characteristics for designing meaningful clinical studies for LMS patients which will deliver novel therapies and help better understand important biological as well as clinical questions.
